# Transboundary Diseases and One Health Approach Implications for Global Health Threats, with Particular Interest in Conservation and Bioterrorism

**DOI:** 10.3390/pathogens14121193

**Published:** 2025-11-22

**Authors:** Massimo Giangaspero, Salah Al Mahdhouri, Sultan Al Bulushi, Metaab K. Al-Ghafri, Pasquale Turno

**Affiliations:** 1 Parliamentary Assembly of the Mediterranean, Center for Global Studies, 47890 City of San Marino, San Marino; 2 Faculty of Veterinary Medicine, University of Teramo, 64020 Teramo, Italy; 3 Environment Authority, Muscat 100, Oman; salah.almahdhori@ea.gov.om (S.A.M.); sultan.albulushi@ea.gov.om (S.A.B.); mataab.alghafri@ea.gov.om (M.K.A.-G.); 4 Independent Researcher, 00189 Rome, Italy; paturno@gmail.com

**Keywords:** bioterrorism, conservation, One Health, transboundary animal diseases, zoonoses

## Abstract

Among animal diseases, those characterized with transboundary potential enhance their interconnection to the One Health principle. Zoonoses with a higher capacity to spread compared to other diseases with a lower level of transmissibility multiply their potential impact on human populations. The routes and speed of transmission and virulence may also increase the impact on animal health in the zootechnic sector and in wild animals. This risk, especially in endangered species, has the potential to alter biodiversity, negatively affecting the environment. The characteristics of these pathogens represent a global health danger that requires knowledge and the capacity for prevention and control, considering the possibility of natural outbreak occurrence together with the deliberate use of such pathogens as biological weapons for terrorist attacks. Animal pathogens, particularly those with zoonotic potential, have long been considered for use in bioterrorism. International conventions prohibit the use of microbiological and toxin weapons. Furthermore, recent European legislation has also addressed the potential misuse of animal pathogens in bioterrorism. In this context, the Parliamentary Assembly of the Mediterranean (PAM) and its Center for Global Studies are committed to preventing global health threats by promoting transboundary cooperation, especially through a One Health approach that links human, animal, and environmental health. In the face of future emergencies, PAM is also committed to promoting greater information sharing for harmonized legislative frameworks and equitable access to resources, to strengthen the resilience of global health systems, especially in developing countries. In both the past and recent history, various outbreaks have been attributed to proven or alleged bioterrorist attacks targeting human or animal populations. This study discusses the general characteristics of several relevant transboundary diseases. Paying high attention to One Health is of utmost importance. However, for a full understanding, it is necessary to consider all related aspects and implications.

## 1. Introduction—Towards the One Health Approach

The expansion of global trade, human mobility, and agricultural production has accelerated the spread of transboundary diseases, making them one of the most critical challenges to human security and international stability [1]. By definition, these diseases spread beyond national borders, moving through human populations, animal reservoirs, and ecological systems. Their impact is not only biomedical but also socio-economic, as proven by the COVID-19 pandemic, recurring outbreaks of avian influenza, and the persistent crisis of antimicrobial resistance [2]. Such phenomena demonstrate how health threats are shaped by globalization, climate change, and geopolitical tensions and why responses rooted in isolated sector-specific strategies remain inadequate.

The One Health approach offers a conceptual and operational framework for addressing this complexity, recognizing the interdependence of human, animal, and plant health, food security, and environmental sustainability [3]. Implementing this approach helps to better identify, prevent, and respond to health threats in an integrated manner. One Health is becoming an increasingly frequent topic invoked in scientific and policy discourse, but the approach is still often underutilized in current practice [4,5]. This is attributable not only to fragmented governance but also to the inherent challenge of coordinating expertise across highly diverse disciplines, where no single actor can command complete knowledge. It is therefore necessary to make concrete efforts to embrace this principle. Although the task is undeniably challenging given the complexity of the framework and concerns that acquiring knowledge across so many interconnected fields may be unattainable, it remains essential to acknowledge the fundamental importance of multidisciplinary collaboration.

At the international level, several international organizations, including the World Health Organization (WHO), the Food and Agriculture Organization (FAO), and the World Organization for Animal Health (WOAH), strongly endorse this approach as a cornerstone of global preparedness. Within the European Union (EU), a dedicated One Health task force was recently established [6], representing a significant institutional innovation. This initiative unites five key EU agencies: the European Centre for Disease Prevention and Control (ECDC), the European Chemicals Agency (ECHA), the European Environment Agency (EEA), the European Food Safety Authority (EFSA), and the European Medicines Agency (EMA). The task force aims to foster cross-disciplinary collaboration to support effective implementation of the One Health agenda in Europe [7]. Planned to operate from 2024 to 2026, it focuses on five objectives: coordinating application of the One Health framework; aligning research efforts under a unified agenda; enhancing knowledge and expertise in the field; improving communication and stakeholder engagement; and building partnerships through collaborative action. By reinforcing the scientific foundation of One Health, promoting integrated guidance and risk assessments, and advancing specialized skills—particularly with the involvement of veterinary professionals—this collaborative, multi-sector approach is expected to reduce the impact and cost of health threats, prevent emerging risks, ease human pressures on the environment, and safeguard essential societal needs such as food security and a healthy environment.

Comparable initiatives have also emerged in other regional contexts. The Parliamentary Assembly of the Mediterranean (PAM) has integrated the One Health approach into its long-standing work on human security and health. Among PAM’s One Health priorities, several topics have been identified: advocacy for global health and immunization (including partnership with Gavi, the Vaccine Alliance), food safety, bioterrorism, animal health and welfare, and protection of the environment and biodiversity. Evaluation of the control and prevention of transboundary, emerging, and zoonotic diseases—including strategies involving research on new vaccines—forms part of the efforts that scientists and health stakeholders must consider in addressing threats of cross-border health crises in the Mediterranean region and elsewhere amplified by climate change and globalization [8]. Within the One Health approach, animal health plays a particularly important role, closely tied to welfare, food safety, and public health. The European Union’s ‘Farm to Fork Strategy’ reflects this interdependence, seeking to integrate welfare and safety considerations across the food chain [9]. Yet stress factors in food-producing animals, especially during transport, reduce welfare and increase vulnerability to transmissible disease, with associated risks of zoonotic spread and antimicrobial resistance. These challenges are especially pronounced in developing countries, where inadequate veterinary and food safety infrastructures further compromise resilience to transboundary threats [10,11]. Finally, deliberate threats such as bioterrorism and agroterrorism complicate the landscape of global health security [12]. Despite stringent international rules, the risk remains. Recent suspicions about Russia’s use of banned chemical weapons in the conflict against Ukraine [13], even if this is not a case of bioterrorism, serve as a contextual illustration of how international norms can be violated in modern conflicts. This reinforces the importance of remaining vigilant in the biological domain as well.

Accordingly, in the One Health framework, key research questions remain open, such as “How can the One Health approach be effectively operationalized to strengthen the prevention, control, and management of transboundary diseases, and what are the implications for global health security?” and other interrelated additional questions: “In what ways do transboundary diseases illustrate the necessity of a One Health approach?”; “How effectively is One Health integrated into international and regional strategies?”; “What institutional and scientific gaps hinder its implementation?”; “How can the integration of animal health, welfare, and food safety reduce the risks of zoonoses and antimicrobial resistance?”; and “What policy and research priorities should be advanced to consolidate One Health as a framework for global health governance?” It is clear that deep reflection and research are needed to find sustainable solutions. Considering the One Health principle in a step-by-step approach is a rational strategy.

Preventing global health threats is also within the mandate and scope of the activities of PAM and its Center for Global Studies (CGS). PAM has always been deeply invested in promoting transboundary cooperation in the health sector and has stepped up its efforts during the COVID pandemic to address global health systems’ vulnerability to emerging diseases. Moreover, a One Health approach has also proven to be fundamental in preventing related global security challenges, such as chemical, biological, radiological, nuclear, and explosive (CBRNe) threats, bioterrorism, and agroterrorism, as shown in the 2024 PAM-CGS report “CBRNe, Bioterrorism, and Agroterrorism: The Impact on Human Security and Safety”, highlighting their potential to destabilize societies and endanger human security [14]. Today, providing policymakers with up-to-date information is crucial, as the international community must strengthen its collective detection and response systems—for which international cooperation has proven to be crucial in the past—while also ensuring equitable access to resources, especially for developing countries. However, all of this might not be sufficient, as new emergencies ought to be treated with a One Health approach. In this context, in addition to information sharing and cooperation, it is crucial to develop harmonized and coherent legislative frameworks. PAM is engaged in increasing awareness of the global health threats of humans, animals, and biodiversity, utilizing objective and science-based consideration of the different aspects and potential implications of the One Health framework for improving scientific capabilities and also encouraging cooperation between universities, research centers, and state and non-governmental stakeholders, coherently with PAM’s One Health program, which focuses on the observation of human, animal, and environmental health interdependence.

This article aims to underline the importance of One Health-related topics like conservation and bioterrorism. In the present article, the general impact of transboundary animal diseases is examined to illustrate their economic, social, and health consequences, focusing on Foot and Mouth Disease (FMD) as a case study linked also with a high risk of decreased biodiversity. Furthermore, the connection between transboundary diseases and bioterrorism is explored through an analysis of selected historical and contemporary cases—including Rinderpest, African Swine Fever (ASF), Plague, and Anthrax—that highlight both the natural and deliberate aspects of cross-border health threats.

More research and policy enforcement is necessary for a more coherent and robust application of the One Health framework as a means of enhancing resilience to global health threats.

## 2. Transboundary Animal Diseases: General Impact and Conservation

To achieve sustainable health for humans, animals, and the environment, the One Health approach encourages active collaboration across multiple fields, including environmental sciences, veterinary practice, and human healthcare. In this framework, wildlife conservation plays a crucial role. In the present article, out of the selected transboundary diseases, only two are considered zoonotic (Plague and Anthrax), with direct animal and public health implications, clearly requiring the One Health approach. Meanwhile, the other three pathogens specifically affect animals, and their high rapid spread and indirect negative influence on public health (affecting food security) mean they cannot be considered as a sectoral health problem, thus they also require the One Health approach. Furthermore, risks to animal health when affecting endangered wild fauna, and thus representing risks of reducing biodiversity, imply the consideration of conservation in the One Health framework.

**Figure 1:**
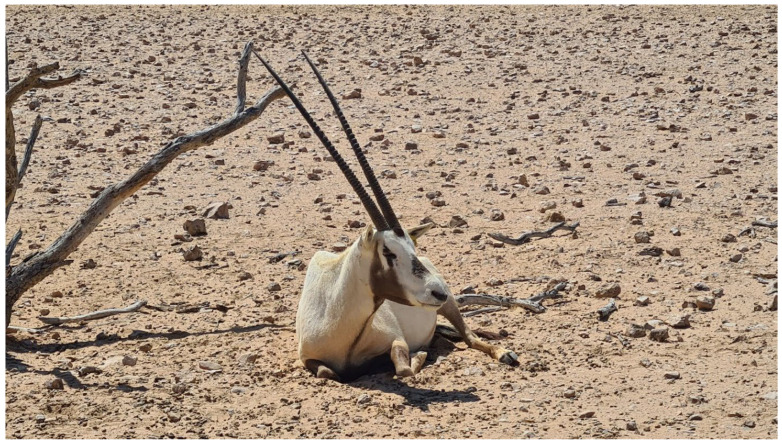
Arabian oryx (*Oryx leucoryx*) at Al Wusta natural reserve, Oman.

Globally, growing awareness of the importance of the environment has prompted many countries to protect wildlife and ecosystems, primarily through anti-poaching measures and the establishment of national parks or other protected areas. A clear example is the Sultanate of Oman, recognized for its rich biodiversity, with emblematic species such as the Arabian leopard (*Panthera pardus nimr*, Hemprich and Ehrenberg, 1833) and the Arabian oryx (*Oryx leucorix*, Pallas, 1777) (Figure 1), which has made significant progress in protecting its wildlife and natural habitats over recent decades. The most important progress was the continued creation of new protected areas for wildlife, distributed in all the regions of the Sultanate: Al Jabal Al Akhdhar reserve, important for the Lappet-faced vultures or Nubian vultures (*Torgos tracheliotos*, Forster, 1791); Al Saleel reserve, located in northern mountains; others along the coast of the Sea of Arabia, such as Ras Ash Shajar reserve and Qurm reserve, sites for many migratory bird species; Ras Al Hadd and Ras Al Jinz reserves, sites of utmost importance for the reproduction of the green turtles (*Chelonia mydas*, Linnaeus, 1758); Jabal Samhan reserve and Khor Taqah sanctuary, the main sites of the Arabian leopard; and the Arabian Oryx Sanctuary. The Oryx Sanctuary (formerly known as Al Wusta Wildlife Reserve—WWR), established under the Royal Diwan’s patronage, as part of its early conservation efforts, was the first wildlife reserve in Oman and is still the most relevant protected area created in the country. It was established in 1980 for the reintroduction and breeding of the Arabian oryx. In 1994, the Arabian Oryx Sanctuary was inscribed in the list of the United Nations Educational Scientific and Cultural Organization (UNESCO). Several studies have been conducted to investigate and provide accounts of the environment, ecology, wild fauna, and vegetation of the Sultanate. Furthermore, environmental laws and legislation in the environmental interest were issued and enforced, undertaking environmental control and inspection, including specific measures against illegal hunting.

More recently, Oman established the Environment Authority and launched its “Vision 2040” strategy, aiming to position the nation among the world’s top 20 nations in wildlife protection. In addition, as in other countries, efforts have been made to prevent illegal hunting, and laws have been strictly enforced. However, although poaching remains a major risk for conservation (in Oman, it led to the extinction of the Arabian oryx in the wild), animal diseases must be carefully considered, including their potential transmission from domestic animals. Many transboundary diseases represent a serious risk for wild animals. Among them, Foot and Mouth Disease (FMD) is certainly one of high importance. Such dangers highlight the scale of challenges that conservation authorities face in protecting wild fauna, particularly vulnerable and endangered species such as the Arabian oryx, a national symbol representing a remarkable reintroduction effort.

### Impact of Foot and Mouth Disease (FMD)

FMD is a highly contagious, transboundary viral disease that severely impacts global livestock production and poses a significant threat to food security. It affects numerous animal species, including humans in rare cases, and has far-reaching economic implications. Global losses due to FMD are estimated at USD 7.6 million annually. The disease is endemic in regions such as Africa, Asia, the Middle East, and South America. A notable outbreak in the United Kingdom in 2001 resulted in economic losses totaling £8 billion (about USD 10.7 billion), primarily due to mass culling of livestock. The virus subsequently spread to France, Ireland, and the Netherlands. Between March and October 2001, 2000 outbreaks were reported in the UK and 6.5 million animals were killed. Cumbria, a northwestern county in England, was especially affected. In February 2001, the region became a vast cemetery. The Watchtree stone memorial was erected to honor animals killed and buried during the 2001 FMD epidemic at Great Orton, now known as ‘the killing field’. In Japan’s southern Prefecture of Miyazaki, a severe FMD outbreak occurred in 2010 [15]. About 290,000 animals, including highly valuable Wagyu beef cattle, were culled, causing severe economic damage and requiring the mobilization of local and national staff as well as the army. National mourning was declared, and a memorial building was constructed in Miyazaki. In Ethiopia, FMD caused an estimated USD 14 million in losses during 2005–2006, significantly impacting the country’s livestock trade with Middle Eastern and African nations. In India, the disease causes annual financial losses of approximately INR 18,000 crores (1 crore INR ≈ 116,279 USD), largely due to a drastic decline in milk production—up to 80% in affected dairy animals. It has been reported that 3508 million kilograms of milk were lost due to FMD, a reduction that may contribute to child malnutrition in low-income farming households that rely on dairy as a primary nutritional source [16].

This last aspect is particularly imperative when considering the overall risk posed by the disease. In addition to the evident impact on animal health and economic losses in livestock production, child malnutrition may be considered a substantial indirect impact on public health. Meanwhile, direct human infection is rare, with only about 40 confirmed cases having been reported worldwide [17]. In humans, clinical symptoms of Foot and Mouth Disease (FMD) may include fever, headache, general discomfort, fatigue, sore throat, pharyngitis, loss of appetite, vomiting, tachycardia, painful ulcerative lesions in the oral cavity, and tingling blisters on the hands and feet. Oral lesions were documented in a 5-year-old child after consumption of raw milk from an infected cow. Vesicular lesions were also observed on the palmar surface of a veterinarian’s finger during an outbreak investigation in cattle [16].

However, from the One Health perspective, another major impact of FMD is the potential to affect the environment by altering biodiversity, particularly through the high risk it poses to endangered and vulnerable species. Such species are rare in the wild, and contact with FMD-infected domestic animals in shared grazing areas may pose an epidemiological risk. This risk is increased when wild animals are reared in captivity for reproduction and repopulation purposes, since higher population densities enhance pathogen transmission opportunities. For example, the Arabian sand gazelle (*Gazella marica*, Thomas, 1897) is considered vulnerable, with an estimated wild population of about 2150 mature individuals [18]. Significantly more are held in captivity centers, such as in Oman and Abu Dhabi [18]. In an FMD outbreak that occurred in the U.A.E. in 2013, the mortality in this species was very high, with deaths accounting for 663 animals, out of a population of 1095 gazelles (60.5%) [19]. Similarly, the Arabian oryx (*Oryx leucorix*) is another important species threatened with extinction, with very few individuals in the wild and about 6000 living in sanctuaries [20]. In January 2025, a severe FMD outbreak occurred in the Oryx Sanctuary at Al Wusta Wildlife Reserve in the Sultanate of Oman. Out of a total population of 669 animals present at the beginning of the outbreak, 226 (33.78%) oryx died—the deadliest event ever reported in the species since its reintroduction from extinction in the wild in 1972 [21].

## 3. Transboundary Animal Diseases and Bioterrorism

Animal pathogens have been considered for bioterrorism, especially those possessing zoonotic potential. Infectious disease control laws thus have specific indications for zoonoses. The World Organization for Animal Health (WOAH, originally established as the *Office International des Epizooties*—OIE) places a significant emphasis on the control of zoonotic diseases. Within the WOAH Terrestrial Animal Health Code, zoonotic potential is a key criterion for including diseases on the official list. A disease must pose a serious risk to human health, beyond artificial or experimental conditions, to qualify. In the context of European legislation, Regulation (EU) 2016/429 of the European Parliament and Council, that was adopted on 9 March 2016 and is commonly referred to as the ‘Animal Health Law’, came into effect in April 2021 [22]. This regulation outlines disease-specific rules for the prevention and control of transmissible animal diseases. According to Article 5 of the regulation (Listing of Diseases), only one zoonosis—highly pathogenic avian influenza—is specifically named among the five priority diseases. However, Annex II, which provides a more comprehensive list, includes several additional diseases, about half of which are zoonotic.

Regarding bioterrorism, ideal features of a biological agent intended for use against animals or humans include high infectivity, strong virulence, absence of available vaccines and treatment options, selection and production of resistant strains, and the presence of an effective delivery system allowing rapid spread and maximum impact on animal and/or public health, potentially leading to public panic and social disruption. For military applications, stability of the weaponized agent (retained infectivity and virulence over extended storage periods) may also be a desirable attribute.

International conventions prohibit the use of biological weapons. Also, most recent European laws take into account the potential use of animal pathogens in bioterrorism. At the international level, bioterrorism is regulated by two key legal instruments. The first is the Geneva Protocol, signed in 1925 in Switzerland, which prohibits the use of bacteriological and toxin-based weapons in international armed conflicts. Later, the Biological Weapons Convention (BWC), opened for signature in London, Moscow, and Washington on 10 April 1972, went further by banning the development, production, and stockpiling of biological and toxin weapons and by mandating the destruction of existing stockpiles. More recently, European Union Regulation (EU) 2016/429 [22] explicitly refers to bioterrorism. Article 5 (Disease Listing) states that a disease must be included in the list if it meets all criteria related to transmissibility, susceptibility, impact on animal or human health, diagnostics, and mitigation measures. In addition, at least one further criterion must be satisfied, such as the potential to trigger a crisis or to be used in bioterrorism (Art. 5(3)(b)(iv)). This potential is also addressed in Article 7, which defines parameters for disease assessment, including the disease’s profile and its possible consequences (Art. 7(c)).

According to the Centers for Disease Control and Prevention (CDC) in the United States, biological agents are classified into three categories [23]. Category A includes agents that are easily disseminated, cause high mortality rates, and have the potential for a major public health impact. They may also cause panic and social disruption, requiring special preparedness. Examples include *Bacillus anthracis* (Anthrax), *Yersinia pestis* (Plague), *Variola major* (Smallpox), and various viral hemorrhagic fevers. Category B includes agents that are moderately easy to disseminate and cause relatively low morbidity and mortality. Examples include *Brucella* spp. (Brucellosis), *Salmonella* spp. (food safety threats), *Coxiella burnetii* (Q fever), and *Vibrio cholerae* (water safety and food threats). Category C includes emerging pathogens that could be engineered for mass dissemination due to their availability, ease of production, and potential for high morbidity and mortality. This group includes viruses such as Nipah virus and Hantavirus.

The historical use of bioterrorism is widely debated. In both past and recent history, various outbreaks have been linked to proven or alleged bioterrorist attacks on humans or animal populations. The use of biological agents as weapons has long been surrounded by a mix of historical accounts, allegations, and myths [24]. Several events throughout history have raised suspicions regarding the intentional spread of disease as a tool of warfare or sabotage, though clear evidence has often been lacking. In many cases, claims of bioterrorism have arisen in politically tense contexts, sometimes serving as accusations between rival nations [25]. For example, certain outbreaks of animal diseases, such as African Swine Fever or Rinderpest, have been regarded by some countries not only as natural events but also as potential deliberate acts intended to cause economic disruption and social unrest [26,27].

While some historical episodes have been investigated under international conventions such as the Biological Weapons Convention (BWC), most of the suspected cases remain unconfirmed or lack conclusive evidence, blurring the line between genuine biological attacks and speculation. Nevertheless, these instances have contributed to growing international awareness of the threat posed by biological weapons to both in human and animal populations. In recent decades, fear of agro-terrorism—the deliberate targeting of livestock or crops—has also gained attention. The devastating impact of such attacks on food security, trade, and national economies has made them a strategic concern for both veterinary and public health authorities.

The following sections discuss myths, allegations, and historical and recent events, taking into account relevant examples of important transboundary animal diseases. In addition to FMD, examples include Rinderpest, African Swine Fever, Plague, and Anthrax. These diseases are among the high priority, classified potential bio-terroristic threats by the Centers for Disease Control and Prevention (CDC) [28], the U.S. Department of Health and Human Services (HHS), and the U.S. Department of Agriculture (USDA) [29]. Similar considerations are shared also by other national and international institutions, such as the European Union and the WOAH. For example, in the Animal Health Law of the EU Regulation 2016 429, Rinderpest is included on the base of the criteria set in Article 7, taking into account that the disease has the potential to generate a crisis or that the disease agent could be used for the purpose of bioterrorism [22]. In addition, because of its reach and magnitude, Rinderpest is considered the deadliest cattle disease in history [30]. The considered diseases’ potential as bioterrorist agents is summarized in Table 1.

### 3.1. Rinderpest

Rinderpest, caused by a *Morbillivirus* of the *Paramyxoviridae* family, was one of the deadliest transboundary animal diseases, affecting cattle, buffalo, and various wild ungulates and causing devastating economic and ecological consequences. The virus is relatively resistant and capable of remaining viable for extended periods in chilled or frozen animal tissues. The disease occurs through direct contact or close indirect exposure to infected animals. Clinical signs included fever, nasal and oral discharges, diarrhea, dehydration, and erosions of the oral mucosa, leading to high mortality in susceptible populations.

Historically, Rinderpest had catastrophic impacts. In the late 19th century, a major outbreak in Africa killed an estimated 90% of cattle and large numbers of wild ruminants, contributing to famine and social disruption. The global veterinary response to Rinderpest has deep roots: The world’s first veterinary school was established in Lyon, France, in 1761, specifically to address this disease. A 1920 outbreak in imported animals in Belgium prompted a push for international collaboration, ultimately leading to the foundation of the Office International des Epizooties (OIE) in 1924 (now renamed World Organisation for Animal Health). By the early 1980s, the global spread of Rinderpest had significantly declined, with remaining outbreaks occurring mainly in parts of Africa, the Middle East, and India. The last reported cases were in Ethiopia, Somalia, and India in 2001, and an investigation continued in Somalia until 2007. Rinderpest was officially declared eradicated worldwide by the OIE on 25 May 2011. It took over 250 years from the founding of the first veterinary school to achieve global eradication. This was a landmark achievement in veterinary medicine and international cooperation, second only to the eradication of smallpox in humans.

In parts of Asia, legends suggest that invading armies may have used Rinderpest as a biological weapon. The “weapon” was said to be herds of Grey Steppe oxen, which, although resistant to the virus themselves, silently spread it over time. These animals reportedly triggered devastating epidemics in buffalo and cattle of targeted communities, resulting in societal collapse—from famine and abandoned fields to the overthrowing of local authorities—making conquest easier for invading forces. Despite such myths and its official eradication, Rinderpest is still treated as a high-priority disease. In fact, it appears first on the list in Annex II of the EU Animal Health Law (Regulation 429/2016) [22]. There are several reasons why authorities continue to be vigilant about Rinderpest. Concerns remain about the potential misuse of preserved virus stocks, since laboratories worldwide retained samples for research purposes even after eradication. Known viral stocks declared to the WOAH are detained in China, Ethiopia, France, Japan, and the USA, but there is also concern about unknown or undeclared samples being held elsewhere. Another risk is the possibility of reconstructing the virus through synthetic biology using previously sequenced genomes. In June 2019, the Pirbright Institute in the UK, home to the World Reference Laboratory for Rinderpest, announced the destruction of all remaining Rinderpest virus stocks. Their policy was to “sequence and destroy”: genomes were fully sequenced before elimination to allow for future reconstitution if necessary [32].

Additionally, Rinderpest is considered a potential threat in the context of agro-terrorism. The deliberate release of Rinderpest could have devastating consequences, given the lack of immunity in current livestock populations and the discontinuation of routine vaccination after eradication. The disease is largely unknown to many livestock producers and is not often considered by veterinarians, making early detection difficult. Clinical similarity with forms of Bovine Viral Diarrhea–Mucosal Disease (BVD–MD) can lead to confusion and delay diagnosis. As a result, the virus could circulate undetected for some time before being identified, thus increasing the potential impact in the affected area.

Despite eradication, the possibility of accidental or deliberate release remains a concern, highlighting the need for vigilance in biosecurity and international cooperation. Therefore, interest in the disease remains strong, as shown by the launch of a Rinderpest recognition course in February 2020 by the Food and Agriculture Organization (FAO), with support from the OIE and the U.S. Defense Threat Reduction Agency [33]. This initiative reduces the risk of accidental release and addresses global concerns about bioterrorism. The success of Rinderpest eradication demonstrates both the potential of coordinated international action and the ongoing responsibility for preventing re-emergence, whether natural or deliberate. The case of Rinderpest underscores how eradicated diseases may still pose biosecurity challenges due to preserved materials, making them relevant to discussions of bioterrorism.

### 3.2. African Swine Fever (ASF)

The causative agent of African Swine Fever belongs to the genus *Asfivirus*. The virus is highly stable in the environment and can persist for long periods in pork products, carcasses, and processed meat, facilitating its spread through trade and food waste. Transmission occurs through direct contact between pigs, ingestion of contaminated products, fomites, and biological vectors such as soft ticks of the genus *Ornithodoros*. ASF is characterized by very high mortality, often reaching 100% in acute cases, and there is currently no effective vaccine. The disease has devastating consequences for pig production, food security, and international trade, making it one of the most feared transboundary animal diseases worldwide. Endemic in sub-Saharan Africa, ASF is a significant threat to pig production, carrying heavy socio-economic consequences. Initially reported in Kenya in 1907, ASF demonstrated its ability to spread beyond its original endemic zones. For example, the virus reached Portugal multiple times, in 1957, 1960, and again during the 1970s to 1990s. The 1970s also saw the spread of ASF to the Caribbean and South America. One of the most notable outbreaks occurred in Cuba in 1971, leading to the culling of approximately 500,000 pigs, representing nearly a third of the island’s total pig population. This drastic measure halted pork production—a staple of the Cuban diet—for several months. Subsequent outbreaks were recorded in the Dominican Republic (1978) and Haiti (1979). Brazil faced an outbreak in 1978–1979, which was successfully eradicated by stamping it out, allowing Brazil to reclaim its ASF-free status by December 1984, after reporting 224 outbreaks. Based on the designation and surveillance of infected zones, with control of pig movements and detailed epidemiological investigation, and tracing of possible sources (upstream) and possible spread (downstream) of infection, the stamping-out programs were applied to deliver rapid slaughtering of all pigs within epidemiological units, proper disposal of cadavers and litter, followed by thorough cleaning and disinfection.

The 1971 Cuban outbreak, the first recorded ASF incursion in the Western Hemisphere, was described by the FAO as the “most alarming event” of that year. It sparked suspicions of deliberate introduction and became one of several Cuban accusations of biological warfare by the United States. Over the decades, Cuban authorities have accused the U.S. of conducting at least 24 biological attacks since 1962, though most claims remain informal and unproven. These allegations include one suspicious event, ten disease outbreaks, and one insect infestation (between 1964 and 1997), attributed to the Central Intelligence Agency (CIA) or U.S.-backed operatives [25]. Only one incident led to an official complaint under Article V of the 1972 Biological and Toxin Weapons Convention (BTWC). In 1997, Cuba submitted a formal grievance to the UN Secretary-General, accusing the U.S. of deploying *Thrips palmi* (an agricultural pest) as a biological weapon in 1996. A consultative meeting was held in Geneva (25–27 August 1997), but the final report concluded that no definitive evidence supported the allegation [34]. However, the 1971 ASF incident continued to attract attention. In 1977, the *San Francisco Chronicle* published articles suggesting U.S. CIA involvement in the introduction of the virus to Cuba [35]. Based on a four-month investigation by *Newsday*’s journalists [36], including interviews with U.S. intelligence sources, Cuban exiles, and scientists, journalists reconstructed the alleged chain of events. According to the report, the virus originated at Plum Island, near Long Island, at the Fort Terry U.S. Army Chemical Corps base, previously used for researching animal infectious diseases. Early in 1971, an unnamed U.S. intelligence agent allegedly received a sealed container with the ASF virus at Fort Gulick (a U.S. Army base and CIA training center in the Panama Canal Zone). The virus was then transferred to Cuban exiles aboard a fishing vessel off the coast of Bocas del Toro, Panama, and taken to Navassa Island, a U.S. territory. From there, in late March 1971, the virus container reportedly reached southern Cuba, near Guantanamo Bay, where it was handed over to local operatives. On 6 May 1971, the first ASF cases appeared near Havana. This alleged reconstruction of the events, though formal proofs or scientific evidence could not be found, was retained in the archives of the U.S. Intelligence Agency and related newspaper clippings were approved for release after analysis and assessment by the Remote Desktop Protocol, and documents made available through the Freedom of Information Act (FOIA) at the CIA Remote Reading Room [37,38].

ASF has long been regarded as a potential bioterrorist agent because of its severe economic consequences, lack of vaccines, and the difficulty of controlling outbreaks once introduced [12]. The intentional release of ASF could cause catastrophic damage to the swine industry of any country, leading to mass culling, trade restrictions, and long-lasting social and economic disruption. Since 2018, outbreaks in China have resulted in the loss of millions of pigs—the largest animal disease outbreak in modern history—with severe repercussions for global pork markets and food prices. The ASF crisis has continued to deepen, with the worst forecasts suggesting pig herds have declined by 35% or up to 200 million pigs lost through infection, slaughter to control the disease, and reduced breeding capacity [39]. An industry analysis indicated that between 2017 and 2020, the total number of pigs raised decreased by almost 125 million in China, a decline of ~28.6%. Much of that loss was attributable to ASF [40]. The uncontrolled spread of ASF in several Asian countries has demonstrated the vulnerability of global food systems and the enormous challenge of containing the virus once it becomes established in wild boar populations. In Europe, ASF has affected both domestic pigs and wild boars, with persistent outbreaks reported in Eastern and Central Europe despite strict control efforts. Wild boar populations represent a significant reservoir of the virus, complicating eradication efforts and maintaining the risk of spillovers to domestic herds. Illegal movement of pigs and pork products has also contributed to the transboundary spread of the disease, highlighting gaps in biosecurity and regulatory enforcement. The absence of effective treatments and vaccines means that control relies heavily on preventive measures such as strict biosecurity, culling of infected animals, and movement restrictions. These measures are difficult to sustain over long periods, particularly in resource-limited settings, and they impose heavy economic and social costs. Given its devastating impact, potential for deliberate introduction, and difficulty of control, ASF exemplifies the type of disease that could be exploited in bioterrorism, making it a critical concern for both veterinary and public health authorities. ASF highlights the importance of global preparedness, early detection systems, rapid response capabilities, and continued investment in vaccine research and novel control strategies.

### 3.3. Plague

Plague is caused by the bacterium *Yersinia pestis*, transmitted primarily through fleas, direct contact, and inhalation. The disease presents mainly in two forms, bubonic and pneumonic. The bubonic form has a case fatality rate of 30–60%, while the pneumonic form is invariably fatal if left untreated.

*Yersinia pestis* was responsible of the infamous “Black Death”, one of the most devastating epidemics in history [41]. This pandemic emerged from the intersection of two expanding worlds: the Mongol Empire and Genoese trade routes. By 1279, the Mongol Empire stretched from China through Central Asia to Iran, Iraq, and most of Russia [42]. Its vast reach enabled efficient communication and trade networks. As global trade expanded, *Y. pestis* was able to traverse long distances, overcoming the geographical barriers and sparse populations that had previously prevented widespread outbreaks. In 1266, the Mongols granted the Genoese permission to establish a trading post in Caffa (modern-day Feodosia) on the Crimean Peninsula [43]. By the 14th century, Caffa had become a thriving port city of around 8000 inhabitants [42]. In 1343, tensions flared in the nearby town of Tana, where Italians killed a Muslim resident. Facing retribution from the Mongols, the Italians fled to Caffa, which was then placed under siege [44,45]. By 1346, Plague had appeared on the western shores of the Caspian Sea [44,46,47]. A year later, it reached Crimea. Mongol soldiers began dying in large numbers from the mysterious illness, and while obliged to abandon the siege, they resorted to hurling the infected corpses over the city walls using catapults, a grim early instance of biological warfare [45]. While historical records and narratives are controversial, like in this case with the plausible though unlikely occurrence of catapulting infected corpses during the siege [45,48], there are chronological and geographical links between a military conflict and a disease outbreak, a favorable epidemiological context for further spread. In the spring of 1347, with the Mongol army decimated, the Genoese escaped by sea, inadvertently spreading the Plague westward. In early summer of 1347, ships departing Crimea passed through Byzantium (modern-day Istanbul), where the Plague claimed 90% of the city’s population by autumn [49]. The disease followed major trade routes and entered the European historical record when it reached Sicily. From Byzantium, twelve Genoese galleys reached the port of Messina, on the coast of Sicily, in October 1347 [50]. As accurately described by a local Franciscan Friar, Michele da Piazza, people began to fall ill. The friar described “a sort of boil, the size of a lentil, erupted on the arm, then the victims violently coughed up blood, and after three days of incessant vomiting for which there was no remedy, they died. And with them died everyone who had talked to them, but also anyone who touched their belongings” [51,52], a clinical description compatible with a hypervirulent pathogen such as *Y. pestis*. From December 1347, the Plague swept across Europe, reaching Sweden by December 1350 [49,53]. Known as “*La Moria Grandissima*”, the Black Death killed an estimated 75 million people across Europe in just one decade. The pandemic reduced Europe’s population by approximately one-third, and population levels did not fully recover for 400 to 500 years [54,55,56]. Sporadic outbreaks continued for another 300 years and contributed to the decline of the feudal system [56,57]. In addition to the medieval pandemic (1346–1400), two other major Plague pandemics are recorded in history. The Justinian Plague (540–590 AD), which may have contributed to the fall of the Roman Empire, caused an estimated 10,000 deaths per day, totaling around 100 million deaths (about 50–60% of the population) [55,56]. The Great Plague of London (1665) then killed approximately 100,000 people out of a population of 460,000 [58].

Though often associated with antiquity, Plague remains a concern in some regions. Notable modern outbreaks include the 1994 epidemic in India [59] and other sporadic occurrences [60]. The three biovars of *Yersinia pestis* are still present in different parts of the world. The Antiqua, linked to the Justinian Plague, is found in Africa and Central Asia; the Medievalis, responsible for the Black Death, is present in Central Asia; and the Orientalis, linked to the last pandemic, is now widespread [61]. They do not differ in their virulence or pathology in animals and humans [62,63].

According to the WHO, Plague is found on all continents except Oceania, but most human cases since the 1990s have occurred in Africa [64]. Averages of 2000 cases of human Plague are reported each year worldwide. From 1000 to 5000 human cases and 100 to 200 deaths are reported annually to the World Health Organization (WHO) [60], and probably many cases are undiagnosed. Plague may recur after long inter-epidemic times, as outbreaks may follow quiescent periods of 30 to 80 years. Plague is therefore categorized as a re-emerging infectious disease. In certain countries, the pathogen is endemic. Sporadic cases and outbreaks can be seen in any endemic region. Seven countries have been affected by Plague virtually every year during the last 45 years: the Democratic Republic of Congo, Madagascar, Myanmar, Vietnam, Brazil, Peru, and the USA [65]. The Democratic Republic of Congo, Madagascar, and Peru are the three most endemic countries. In Madagascar, about 300 to 650 human Plague cases are reported per year [66]. Cases of bubonic Plague occur mostly between September and April. However, most cases in the last outbreak in 2017 were pneumonic Plague in previously non-endemic areas, including densely populated coastal urban areas [67]. In other countries, reports in the media are sporadic but not so uncommon. For example, in Kyrgyzstan, in 2013, health officials feared an outbreak of bubonic Plague in central Asia after a 15-year-old boy died from the disease and three more were admitted to hospital, after eating an infected barbecued marmot [68]. In China, in 2014, China Central Television reported the death of a 38-year-old man due to bubonic Plague after feeding his dog a marmot [69].

Human exposure to Plague is not limited to rats and fleas. Other transmission routes include contact with tissues from infected wildlife [57,70], consumption of infected animals (food safety issue) [71], laboratory accidents [72], close contact with pneumonic Plague patients [73], exposure to infected domestic cats [74], and bioterrorism events [75]. Plague is also a modern veterinary concern. In endemic areas like Colorado (USA), public warnings are issued about Plague in wild rodents such as chipmunks and ground squirrels [76]. Domestic cats are particularly susceptible; they can contract Plague through the oral route while hunting infected rodents [77,78]. These infections often present as submandibular buboes, which can be mistaken for abscesses caused by common pyogenic bacteria. For veterinarians, handling infected cats poses a serious risk. Transmission via respiratory droplets can result in septicemic Plague, which may lead to necrosis of extremities due to micro-thrombosis and severe disease progression [76]. Apart from the natural infection occurrence, the disease is also considered important due to the potential use in bioterrorism. Comprehensive accounts are given on the Japanese use of Plague as a weapon in World War II, after 1932, when the Japanese army invested budget and human resources for medical experiments [79]. Plague is classified as category A on the list of Bioterrorism Agents [28], especially taking into account the potential for developing antibiotic-resistant strains, including resistance to streptomycin and multi-drug resistance [80,81,82].

### 3.4. Anthrax

Anthrax, caused by *Bacillus anthracis*, a highly resistant bacterium, occurs via direct contact, ingestion, or inhalation [83]. Infection can result from breathing in spores, consuming contaminated food or water (posing a food safety risk), or through skin exposure when spores enter lesions. Anthrax manifests in cutaneous, gastrointestinal, and respiratory forms. Anthrax inhalation is the most lethal; even with intensive treatment, approximately 45% of patients do not survive [84]. Epidemiology involves both sylvatic (wildlife) and domestic animal transmission cycles [85]. Within the sylvatic transmission cycle, blowflies consuming infected carcasses are thought to serve as key vectors [86]. After feeding, they regurgitate blood onto vegetation while resting, indirectly contaminating the environment and infecting herbivores. Carnivores feeding on infected animals often develop subacute to chronic Anthrax, marked by severe facial swelling and high mortality [87]. In the domestic cycle, livestock become infected through contaminated pastures or water sources [85]. Humans are typically exposed through handling tissues and skins of infected animals [83]. Cutaneous Anthrax in humans can lead to severe complications [84].

Anthrax is widespread globally, and natural outbreaks can be severe. A notable example occurred in Bangladesh between August and October 2010 [88]. Local newspapers reported daily updates with alarming headlines such as “67 More Anthrax Patients Detected” (23 August, *New Age Journal*), “298 People Infected with Anthrax” (5 September), “Anthrax Spread to 6 Districts” (6 September), and “Anthrax Spread in 13 Districts, 534 Affected” (14 September, *The Daily Jonokontho*). Images of infected children published in newspapers (e.g., *The Daily Star*, 6 September, Dhaka) further intensified public fear. By September 26, the worsening crisis prompted a formal request for international aid. The Ministry of Fisheries and Livestock of Bangladesh appealed to the FAO, which responded through the Crisis Management Centre—Animal Health. A mission was carried out from October 23 to November 3. Ultimately, between August and October 2010, Anthrax outbreaks were reported in 15 districts, with 607 human cases and 104 animal cases. As ascertained by the FAO–OIE expert mission, the outbreak originated from a single sick goat, illegally imported from India [89]. According to the investigation, the first human cases (eight people developing skin lesions) were traced to a village of the Shaturia subdistrict, subsequent to the slaughtering of a single sick goat and preparing meat for cooking. The affected animal had not been reported to the local Livestock Officer [89].

A modern example of Anthrax as a bioterrorist agent occurred in the United States in 2001, shortly after the September 11 terrorist attacks [90]. Letters containing Anthrax spores were sent to government officials and media representatives [91]. In total, 22 individuals were infected, including 9 U.S. postal workers, 2 of whom died. On October 2, a newspaper employee in Florida was hospitalized and died three days later from inhalation Anthrax. Then, on October 31, a hospital worker in New York died from inhalation Anthrax. On November 21, a 94-year-old woman in Connecticut also died from the same cause [92]. Despite the relatively small scale of the attack, it led to widespread fear and prompted 32,000 individuals to begin antimicrobial prophylaxis [91], with 10,300 completing the recommended 60-day ciprofloxacin or doxycycline treatment, as spores can remain dormant after the initial infection and later reactivate [93].

Hypothetical deliberate release scenarios have been considered. A simulation study by Costantino et al. [94] examined the impact of an aerosolized *Bacillus anthracis* release over Sydney, Australia (population: 5 million; density: 2000 people/km^2^). Considering meteorological conditions, wind speed/direction, and topography, and using a light aircraft, spores could be dispersed over a 70 km^2^ area. Assuming a release rate of 4 g per second for one hour (with each gram containing approximately one trillion spores), around 500,000 people could be exposed, and more than 350,000 infected. Even with vaccines and antibiotics, fatalities could range from 200,000 to 316,000, depending largely on how quickly countermeasures are deployed (ideally, they should start early, 2 days after release). In another modeled scenario involving the U.S. capital Washington, D.C. (population: 7.7 million; density: 3650/km^2^), the upwind release of 100 kg of Anthrax spores could result in up to 3 million deaths [95].

It should be taken into account that the recent technological advances represent an increased threat of bioterrorism. For example, molecular genetic engineering techniques, such as the clustered regularly interspaced short palindromic repeats (CRISPR) gene editing, might be applied in molecular biology to modify the genomes of living organisms to produce lethal or sub-lethal pathogens with enhanced virulence, as is the case for the potential development of “invisible Anthrax” strains, modified to evade immune responses and render vaccines ineffective [31], as well as natural or engineered strains resistant to antibiotics. The potential application of such new engineering technologies was even evoked by the eminent professor Montagnier (co-discoverer of the HIV virus) in the case of a supposed artificial origin of the severe acute respiratory syndrome Coronavirus 2 (SARS-CoV-2), during the COVID-19 pandemic. Furthermore, the application of nanotechnology and new delivery systems, and especially artificial intelligence, which is currently carefully considered by PAM in consultation with the Counter-Terrorism Committee Executive Directorate (CTED) of the UN Security Council, might represent additional threats.

## 4. Conclusions

The theme of transboundary diseases, their general impact, their potential risk in reducing biodiversity, and their potential use as biological weapons are highly complex and demand a multidisciplinary approach supported by a well-coordinated international surveillance network to effectively address emerging challenges. Multiple factors must be taken into account, including epidemiology, diagnostics, risk assessment, communication, legislation, collaboration, animal health and welfare, and zoonotic diseases. A range of organizations may play key roles, particularly the OIE (within the framework of the WTO Sanitary and Phytosanitary Measures—SPS Agreement), along with WHO, EU, EFSA, and FAO. Zoonoses are probably the most important field linking veterinary and human medicine, demonstrating the necessity of the application of the One Health framework. It has been noted that approximately 75% of new emerging human infectious diseases are defined as zoonotic [96]. Even the EFSA and ECDC annual EU Summary Reports on zoonoses, zoonotic agents, and foodborne outbreaks have been renamed the ‘EU One Health Zoonoses Summary Report’ (EUOHZ), since 2019. In fact, One Health offers a sustainable, long-term solution for the prevention and control of zoonoses, requiring political commitment, intersectoral coordination, and community engagement. Advantages of this approach in tackling zoonoses are still not seemingly being considered, especially in developing countries where transboundary diseases, including zoonoses, have the greatest impact. Additionally, strategies to counter bioterrorism should embrace the One Health principle, emphasizing the need to enhance knowledge and preparedness to ensure efficient prevention and control measures. Similarly, maintaining the delicate environmental balance, safeguarding unique ecosystems, and ensuring the survival of endangered species for future generations require continuous research and strategic management of natural resources.

There is a need to increase research on all One Health-related sectors and implications [97], not limited to zoonoses, food safety, and agriculture, but extended to other topics such as bioterrorism and conservation, to improve the full understanding of this concept. Strengthening information and promoting awareness on One Health is of outmost importance and necessary to achieve consistent application. This may include methodologies for supporting country authorities in assessing their One Health capabilities, strengthening regional epidemic preparedness, in particular against transboundary zoonotic diseases, and reaching compliance with international frameworks [98,99]. Myths, allegations, and narratives cannot be confused with science-based facts, and of course they generate disagreements among researchers supporting antithetical evaluations. However, they are expressions of beliefs present in our actual societal culture and help to maintain attention paid to topics that deserve high consideration and continuous awareness.

Preventing global health threats also remains a key priority for PAM and its Center for Global Studies. Building on its strong track record of fostering transboundary cooperation, PAM continues to advocate for a coordinated international response based on timely information sharing, equitable access to resources, and inclusive policymaking. Yet, as future emergencies will increasingly require a One Health approach, PAM underscores the urgent need for coherent and harmonized legislative frameworks to enhance the resilience of global healthcare systems.

## Figures and Tables

**Table 1:** Bioterrorism considerations on some relevant transboundary diseases classified as high priority by the Centers for Disease Control and Prevention (CDC) [28], the U.S. Department of Health and Human Services (HHS), and the U.S. Department of Agriculture (USDA) [29].

Disease	Bioterrorism Category	Zoonotic Disease	Laboratory Feasibility	Method of Dissemination	Impact
FMD	Tier 1 Agent	Minor	Easily replicated in laboratory	Highly contagious, high risk in non-endemic countries	Severe impact on livestock production and significant threat to food security
Rinderpest	Tier 1 Agent	No	Potential for virus stock production or genome reconstitution via sequencing technologies	Infection of highly susceptible cattle populations	Severe disruption to the cattle industry and economy, with major socio-economic consequence
African Swine Fever	Tier 1 Agent	No	Selection and propagation of highly virulent virus strains	Infection of susceptible animal populations (no need for transporting live infected animals)	Devastating consequences for the swine industry and national economy, with broad socio-economic disruptions
Plague	A	Yes	Potential for developing antibiotic-resistant strains, including resistance to streptomycin and multi-drug resistance	Possible deployment includes infecting susceptible rodent populations	High potential for public health crises, mass panic, and social disruption
Anthrax	A	Yes	Anthrax spores are easy and quick to produce. There is potential for the development of “invisible Anthrax” strains, modified to evade immune responses and render vaccines ineffective [31], as well as natural or engineered strains resistant to antibiotics, including penicillin, amoxicillin, quinolones, and macrolides.	Primarily through aerosolized release	High psychological and social impact due to widespread public fear. Severe burden on healthcare systems, particularly emergency and intensive care units; high lethality in certain clinical forms.
